# Acute hepatitis a virus infection presenting with multiorgan dysfunction: a case report

**DOI:** 10.4076/1757-1626-2-8124

**Published:** 2009-07-30

**Authors:** Abdul Rasheed, Shahzad Saeed

**Affiliations:** Combined Military HospitalRawalpindiPakistan

## Abstract

**Introduction:**

Acute hepatitis due to hepatitis a virus is usually a benign self-limiting disease conferring lifelong immunity. However, few cases have been reported in literature with fulminant hepatitis. We report this extremely rare case with multiorgan dysfunction including liver failure, hepatic encephalopathy, renal failure, pleural effusion, pericardial effusion and hematologic dysfunction as a sequale of this infection in an otherwise healthy male at the age of 18.

**Case presentation:**

An 18 years old Pakistani male presented with two days history of fever, cough, headache and vomiting. His condition gradually deteriorated and on day 7 developed multiorgan dysfunction. Initially Immunoglobulin M anti hepatitis a virus was borderline 1.40 but repeated titers one week later confirmed the diagnosis of acute hepatitis a virus infection.

**Conclusion:**

This original case report highlights the importance of focusing first uncommon manifestations of common illnesses while diagnosing difficult cases. Moreover this case also adds knowledge to the limited available data regarding complications and predictors of prognosis.

## Introduction

Hepatitis A virus has plagued mankind for centuries by causing acute hepatitis associated with significant morbidity and occasional mortality. HAV is a 7.5-kb positive-strand RNA virus of the Picornaviridae family and the only member of the genus Hepatovirus [1]. All four genotypes belong to a single serotype. HAV is spread via the fecal-oral route. The incubation period averages 30 days (range 15 to 49 days). The prevalence of HAV infection varies among countries in Asia [2]. Countries with high endemicity for HAV infection include Pakistan, India, China, Nepal, Bangladesh, Myanmar and the Philippines. Most people in these countries are exposed during childhood. HAV infection usually results in an acute, self-limiting illness and only rarely leads to fulminant hepatic failure [3]. In young children, the disease is often asymptomatic, whereas in older children and adults there might be a range of clinical manifestations from mild, anicteric infection to fulminant hepatic failure. The risk of fulminant hepatitis is high in patients having an underlying chronic liver disease and are aged more than 40 years [4]. This case report describes a young person from a highly HAV endemic area with serologically confirmed acute HAV infection with multiorgan involvement.

## Case presentation

An 18 years old Pakistani male presented with about two days history of intermittent fever with chills, nonproductive cough, generalized headache, nausea and nonbilious vomiting. He vomited thrice on day 1 and five times on the next day. Vomitus contained food particles and was devoid of blood. Clinical examination was unremarkable except raised temperature ranging from 37.22°C (310.4 kelvin) to 39.44°C (312.6 kelvin) with relative bradycardia (pulse ranging from 56/minute to 84/minute). Initial investigations revealed raised serum alanine aminotransferase (2043u/l), low normal platelet count (165 × 10^9^/l) and total white cell count (4.2 × 10^9^/l) with normal differential count and morphology. Other investigations including haemoglobin, malarial parasite slides, bilirubin, alkaline phosphatase, aspartate aminotransferase, albumin, urea, creatinine, electrolytes, plasma glucose, widal test, DIC screening, urinalysis and chest radiograph were within normal limits. He was provisionally diagnosed as a case of anicteric hepatitis with differential diagnoses of malaria and enteric fever due to their high prevalence in the area. He was managed with antimalarial (artemether), third generation cephalosporin (Ceftriaxone) and supportive parenteral fluids. Samples for blood cultures, viral (including hepatitis and dengue) screening, typhi dot, serology for brucella, leptospira, rickettsia and toxoplasma were sent to laboratory.

On day 3, he developed dizziness and unsteadiness of gait and asterixis while fever with relative bradycardia, headache and vomiting continued. CT scan head revealed no abnormality. IgM anti HAV was borderline 1.40 (cut off 1.20). Repeated investigations showed rising serum alanine aminotransferase 3690u/l, prothrombin time 30 seconds (control 12 seconds), PTTK 46 seconds (control 32 seconds), fibrinogen 130 mg/dl, D-dimers >200<400, serum albumin 28 g/l, urea 13 mmol/l, creatinine 266 umol/l, sodium 133 mmol/l potassium 4.8 mmol/l, creatinine kinase 1112 u/l with CK-MB 6.1%, LDH 6130 u/l, AST 66 u/l, haemoglobin 12.8 g/l, total white cell count 11 × 10^9^/l, platelets 116 × 10^9^/l, pus cells(8-10/HPF) & red blood cells (5-7/HPF) seen on urinalysis. Other investigations including ECG, bilirubin, alkaline phosphatase, hepatitis B surface antigen, serology for hepatitis E, C, dengue, brucella, leptospira, rickettsia, toxoplasma and typhoid were normal. Antimalarial (artemether) was stopped when repeated malarial parasite slides were found negative. Vitamin K was added to treatment but his clinical and laboratory parameters continued to deteriorate.

On day 7, fever settled but his blood pressure rose to 170/110 mmHg and became oliguric with 24 hour urinary output falling to 150 ml while investigations revealed urea 14.1 mmol/l, creatinine 1204 umol/l, sodium 127 mmol/l, potassium 4.4 mmol/l, serum alanine aminotransferase 2149 u/l, bilirubin 77 umol/l, alkaline phosphatase 367u/l, prothrombin time 16 seconds, PTTK 39 seconds and fibrinogen 180 mg/dl. Ultrasonography showed bilateral pleural effusion (mild) and renal parenchymal disease with increased echogenicity × normal sized kidneys (Right 11.2 cm, Left 11.6 cm).

Echocardiography showed minimal amount of pericardial effusion with no evidence of tamponade. He was managed with frusemide and haemodialyzed thrice on day 7,8 and 10 via dual lumen catheter in right subclavian vein.

On day 10, he started showing signs of improvement with better control of blood pressure, urinary output improving to 900 ml/24 hours and significant improvement in the levels of serum urea/creatinine as well as the liver function tests etc. Repeated IgM anti hepatitis A virus was positive, while rest of the investigations including blood cultures, serum cryoglobulins, aldolase, rheumatoid factor, complement levels, autoimmune and vasculitic screening revealed no abnormality. Dual lumen catheter was removed post dialysis on day 10.

On day 16, investigations revealed normal serum albumin, coagulation profile, cardiac enzymes, electrolytes and blood counts while levels of serum urea 11.2 mmol/l, creatinine 246 umol/l, alanine aminotransferase 127 u/l, bilirubin 38 mmol/l, alkaline phosphatase 594 u/l were showing gradual improvement.

Follow up on day 23 revealed completely normal clinical and laboratory parameters including renal, hepatic, cardiac, pleural and hematological functions. Monthly follow up during last five months has not shown any evidence of relapse.

## Discussion

HAV infection usually results in an acute, self-limiting illness conferring lifelong immunity and only rarely leads to fulminant hepatic failure. Fulminant hepatic failure occurs more commonly in patients with underlying liver disease; particularly chronic hepatitis B and C infection, advanced age and addiction of intravenous drugs [3-6]. This case is very unusual as there was no pre-existing hepatic or non hepatic illness or other risk factors.

Few cases of acute renal failure and nephrotic syndrome have been reported in the literature in association with HAV infection [7-10]. Acute tubular necrosis was the most common form of renal injury in such patients while in others renal biopsy was suggestive of interstitial nephritis, immune complex mesangial glomerulonephritis [7], and IgA dominant glomerulonephritis. Only once IgA dominant glomerulonephritis was accompanied with cutaneous cryoglobulinemic vasculitis.

This is a truly rare event in which a young patient at the age of 18, experienced multiorgan dysfunction secondary to hepatitis A virus infection comprising of fulminant liver failure, hepatic encephalopathy, acute renal failure, pleural effusion, pericardial effusion and hematological dysfunction within short span of time and without preexisting underlying liver disease.

## Conclusion

This emphasizes the importance of focusing on common illnesses with their uncommon manifestations while searching for solution of various clinical diagnostic mysteries even in the absence of poor prognostic markers.

## Abbreviations

AST: Aspartate transaminase
CK-MB: Myocardial fraction of creatinine kinase
DIC: Disseminated intravascular coagulation
ECG: Electrocardiogram
HAV: Hepatitis A virus
HPF: High power field
Ig: Immunoglobulin
LDH: Lactate dehydrogenase
PTTK: Partial thromboplastin time with kaolin
RNA: Ribonucleic acid
